# Relationship between accelerometer-measured physical activity and depressive symptoms in hemodialysis patients with comorbid diabetes mellitus: a multicenter cross-sectional study

**DOI:** 10.3389/fpsyg.2025.1478765

**Published:** 2025-01-31

**Authors:** Ruiting Liang, Xiaoyu Chen, Gaowa Siqin, Zhixin Zhang, Shumei Zhang, Lihua Li, SarNa Talin, Qi Guo

**Affiliations:** ^1^Department of Rehabilitation Medicine, Shanghai University of Medicine and Health Sciences Affiliated Zhoupu Hospital, Shanghai, China; ^2^School of Sports and Health, Tianjin University of Sport, Tianjin, China; ^3^Department of Cardiovascular Medicine, Inner Mongolia People’s Hospital, Hohhot, Inner Mongolia, China

**Keywords:** accelerometer, depressive symptoms, hemodialysis, moderate-to-vigorous physical activity, type 2 diabetes mellitus

## Abstract

**Objective:**

The objective of this study was to examine the association between accelerometer-derived moderate-to-vigorous physical activity (MVPA) and depressive symptoms in hemodialysis patients with and without type 2 diabetes mellitus (T2DM).

**Methods:**

This cross-sectional study finally included 450 maintenance hemodialysis patients (male 281, average age 62 years) from seven dialysis centers in Shanghai, China. Physical activity (PA) was measured using the triaxial accelerometer (ActiGraph GT3X+, Pensacola, FL, United States). We measured depressive symptoms using the 9-item Patient Health Questionnaire (PHQ-9) (PHQ-9 ⩾ 10). We categorized physical activity into quartile groups (Q1 through Q4), with the first quartile defined as low engagement and the remaining quartiles defined as moderate to high engagement. We used logistic regression and trend test to analyze the relationship between quartile groups and depressive symptoms. The analyses in this study adjusted for a range of confounders.

**Results:**

The prevalence of depression was higher in patients with hemodialysis combined with T2DM (17.2%). In diabetic patients, MVPAQ4 was negatively associated with depression after adjusting for covariates [OR = 0.076; 95% confidence interval (CI) = 0.006–0.955, *p* = 0.046]. However, in non-diabetic hemodialysis patients, no significant association was found between MVPAQ1-Q4 and depression after adjusting for covariates (*p* > 0.05).

**Conclusion:**

Moderate-to-vigorous physical activity was associated with depression in the diabetic hemodialysis group, but not in the non-diabetic group. Further studies are needed to investigate more causal relationships between MVPA and depressive symptoms in patients with T2DM.

## 1 Introduction

Depression is the most commonly occurring psychological complication in patients undergoing maintenance hemodialysis (HD), due to its high prevalence and association with other adverse medical outcomes (Senanayake et al., 2018). Many studies have reported that the prevalence of depression in dialysis population ranges from 13 to 43% (Palmer et al., 2013; Shirazian et al., 2017). The worsening in depression is often influenced by many factors, such as age, education, vascular diseases, and chronic diseases (diabetes, hypertension) (Kimmel and Patel, 2006; Schouten et al., 2019). Furthermore, patients with chronic kidney disease (CKD) are frequently associated with protein-energy depletion and metabolic disorders, which can result in a reduction in muscle mass and a decline in physical fitness (Gordon et al., 2018). Therefore, it is essential to identify depressive symptoms early and evaluate the modifiable factors associated with it.

Physical activity (PA) is defined as any bodily movement produced by skeletal muscles that result in energy expenditure (Colberg et al., 2016). Meta-analyses of prospective studies have shown that PA was associated with lower risk of depression (Pearce et al., 2022). Moderate to vigorous physical activity (MVPA) in PA is known to be a significant risk factor for depression. Consistent evidence suggests that depression rates are lowest among the most physically active fraction of the population (Teychenne et al., 2008). Furthermore, MVPA and depression are mutually related as research has shown that individuals with depression are generally less physically active than those without depression, simultaneously, lower levels of MVPA increase the risk of depression (Park et al., 2024). However, the meta-analyses also acknowledged the limitations of the existing studies: reliance on self-reported measurements, which are prone to bias (Pearce et al., 2022). As several studies have used accelerometers to measure physical activity outcomes in HD, specifically, more population-based data are needed to understand the amount of habitual physical activity in HD and the physical activity characteristics associated with depression (Gomes et al., 2015; Kim et al., 2014; Sember et al., 2021).

Besides the role of MVPA, the diabetes status may also affect depressive symptoms. It is noteworthy that T2DM is a common comorbidity among chronic hemodialysis patients, with a prevalence of 10–40% (Bianchi and Volpato, 2016). Several recent studies have indicated that T2DM (Su et al., 2023) and poor physical activity (Narita et al., 2019) are significant predictors of depression in hemodialysis patients. And previous studies have shown that T2DM is related to depressive symptoms among hemodialysis patients (Cavallo et al., 2022; Ebert et al., 2021), but some individual studies have no association (Cavallo et al., 2022; Kim et al., 2022). Thus, it is reasonable to believe that MVPA and T2DM may interact in some way and in turn have an effect on depression. However, to date the relationship of these three conditions of chronic hemodialysis patients remains controversial. Moreover, although physical impairment, T2DM, and depression were examined in patients with advanced chronic kidney disease (Alshelleh et al., 2022; Wang et al., 2017), no studies have yet investigated the potential relationship among MVPA, diabetes status and depressive symptoms in the patients undergoing hemodialysis using accelerometers.

Therefore, the aim of this study was to investigate the relationship between objectively measured physical activity and depressive symptoms in hemodialysis patients. Based on the above indications, it was hypothesized that the presence of diabetes would lead to poorer physical activity and a high prevalence of depressive symptoms. Among other things, this study focuses on the relationship between moderate-to-vigorous physical activity physical activity and depressive symptoms to provide evidence for clinicians to effectively manage depressive symptoms in hemodialysis patients.

## 2 Materials and methods

### 2.1 Subjects

This was a multicenter cross-sectional study that included patients undergoing hemodialysis from July 2020 to April 2021 at seven hemodialysis centers in Shanghai. Patients were older than 18 years old, had received maintenance hemodialysis for at least 3 months, and were able to provide informed consent. Patient exclusion criteria were as follows: (1) those who were unable to communicate with the researchers or were unable to provide informed consent; (2) those who were unable to measure body composition; (3) those who refused to wear the accelerometer; (4) participants diagnosed with serious mental illnesses such as Alzheimer’s disease and schizophrenia; (5) manifestation of infection, amputation, pulmonary edema, or malignancy; and (6) no blood samples. Subjects’ family members were also informed and gave consent. Subjects could withdraw unconditionally at any time if they felt unwell during the study. After the exclusion of 34 subjects (28 patients with incomplete watch data, 6 without laboratory data), the final sample analyzed was 450 (65 depressed and 385 non-depressed). The study was approved by the Ethics Committee of the Shanghai Medical College of Health Sciences, and the research methodology was conducted in accordance with the principles of the Declaration of Helsinki. In addition, all patients signed an informed consent form before enrollment in this study.

### 2.2 Accelerometer data

We used the ActiGraph GT3X+ to collect physical activity estimates, which included assessing subjects’ walking, running, and daily activities by measuring vertical acceleration or activity counts. Accelerometer data were screened and analyzed using ActiLife software (version 6.0, Pensacola, FL, United States) with a sampling period of 60 s and a sampling frequency of 60 Hz, which was utilized to remove sleep time based on the subject’s sleep log. Subjects wore the accelerometer while awake for 7 consecutive days, except when showering or swimming. They were asked to return the device immediately after the experiment (Trost et al., 2005). We defined SED as 0–100 count/min, LPA as 100–1951 count/min, and MVPA as 1,952 count/min (Pedisic and Bauman, 2015). We defined no-wear time as the number of activities for at least 60 min as 0. In order for the participant’s accelerometer data to be valid for analysis, accelerometer wear for at least 10 h per day was considered a valid wear day, and participants with at least four valid wear days in a week (including at least two full dialysis days and two non-dialysis days) were included in the analysis. We validated wearing time using ActiLife version 6.5.5 and an algorithm developed by Troiano (2006). The physical activity variable of the accelerometer was reported every 10 min and daily averages were reported. In the 10-min round, we allowed a 1- or 2-min break below threshold. Objective physical activity variables included the average daily number of minutes (a) LPA, (b) MVPA.

### 2.3 Depressive symptoms

Depressive symptoms were assessed by the validated Dutch version of the PHQ-9 (Kroenke et al., 2010). The PHQ-9 is a self-administered questionnaire based on the Diagnostic and Statistical Manual of Mental Disorders, Fourth Edition criteria for major depressive disorder (Doering, 2015). It consists of nine items rated on a four-point scale ranging from 0 = “not at all” to 3 = “almost every day”. The PHQ-9 measures depression symptoms, including thoughts about oneself and psychological problems, and somatic symptoms of depression, which include a wide range of bodily sensations that the depressed person perceives as unpleasant or worrisome (Janssen et al., 2016). Response options were used to calculate a continuous total score ranging from 0 (no symptoms) to 27 (almost all symptoms on a daily basis). In the present study, a predetermined cutoff score of ⩾10 was used as a dichotomous scoring system to define clinically relevant depressive symptoms. The assessment scale was completed during the 4 h the patient was on hemodialysis.

### 2.4 Diabetes mellitus assessment

To determine diabetes status, all participants were based on self-reports, and we again carefully checked the diabetes data through electronic medical records. According to the American Diabetes Association 2021 criteria, fasting plasma glucose (FPG) level ≥ 7.0 mmol/L or 2-h plasma glucose ≥11.1 mmol/L during an oral glucose tolerance test (OGTT) or HbA1c ≥6.5% was considered as diabetes (Konopka et al., 2022). We refer to the above information to determine that patients with type 2 diabetes mellitus.

### 2.5 Covariates

All subjects were invited to participate in a face-to-face interview to answer a standardized questionnaire. Baseline socio-demographic characteristics, behavioral characteristics, and chronic disease prevalence were used as covariates. Demographic characteristics included age, gender, marital status, education, and occupation. Behavioral characteristics included alcohol consumption, smoking, sleep duration, and dialysis duration. Comorbidity was assessed using the Charlson Comorbidity Index (CCI). We collected biochemical data including serum albumin, hemoglobin, calcium, phosphate and parathyroid hormone (PTH) within 3 months of the physical assessment. In addition, dialysis adequacy was defined as total urea fraction clearance index (Kt/v).

### 2.6 Statistical analyses

Baseline characteristics of participants were listed according to the categorization of T2DM and depressive symptoms. Continuous variables are presented as mean ± standard deviation (SD) and categorical variables as numbers and percentages. Baseline sociodemographic characteristics were analyzed using *t*-tests, Pearson’s chi-square test, and Mann–Whitney *U* test. Binary logistic regression analysis was used to analyze the relationship between moderate-to-vigorous hemodialysis physical activity and depression in hemodialysis patients in the non-T2DM and T2DM groups. Depression was used as the dependent variable, and the components of physical activity (MVPA) (Q1, Q2, Q3, and Q4) were used as independent variables, and some confounders [age, gender, body mass index (BMI), widowed, living alone, education level, smoking, alcohol consumption, Kt/v, CCI] were adjusted as covariates. Trend test models were used to analyze the relationship between quartiles of moderate-to-vigorous physical activity and various depressive symptoms in the non-T2DM and T2DM groups. All statistical analyses were performed using SPSS V26.0 software, and differences were defined as significant when *p* < 0.05.

## 3 Results

**Figure 1:**
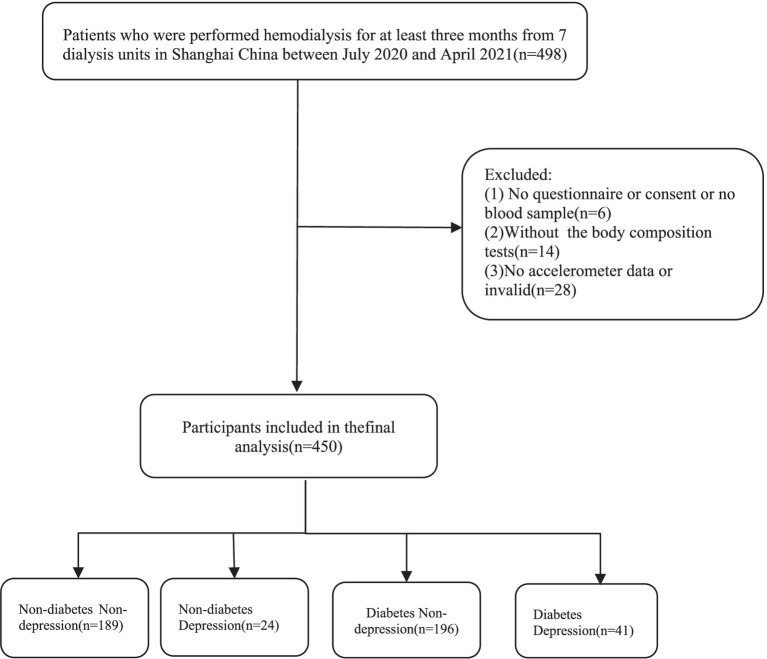
Flow diagram of the study.

Figure 1 shows the grouping process of hemodialysis participants. The final analyzed sample consisted of 450 participants (281 males; mean age: 62 ± 13.2 years), of whom 237 (52.6%) had T2DM and 75 (16.7%) were depressed. Table 1 shows the socioeconomic and health-related characteristics of hemodialysis patients grouped by T2DM and depression. Depressed patients without T2DM were more likely to be widowed compared to patients in the non-depressed group (*p* < 0.05). Hemodialysis patients with T2DM were more male and had shorter life expectancy compared to non-T2DM patients, with a significant difference in CCI (*p* < 0.05, Table 1).

**Table 1:** Regression analysis of moderate to heavy physical activity and depressive symptoms in the non-diabetic and diabetic hemodialysis patients.

Variables	Total (*n* = 450)	Non-diabetes (*n* = 213)		*p*-value	Diabetes (*n* = 237)		*P*-value
		Non-depression (*n* = 189)	Depression (*n* = 24)		Non-depression (*n* = 196)	Depression (*n* = 41)	
Age (y)	62.06 ± 13.2	59.87 ± 15.12	64.21 ± 11.70	0.177	62.82 ± 10.98	67.24 ± 12.58	0.023
Men (%)	281 (62.4)	136 (88.8)	17 (11.2)	0.034	132 (83.5)^a^	26 (16.5)	0.627
BMI (kg/h^2^)	23.3 ± 3.94	22.53 ± 3.31	22.46 ± 4.94	0.933	24.22 ± 4.20^a^	23.01 ± 3.96^b^	0.091
Widowed (%)	81 (18.0)	172 (91.2)	17 (8.8)	0.006	171 (87.2)	25 (12.8)	0.359
Living alone (%)	92 (20.4)	169 (89.7)	2 (10.3)	0.825	176 (86.8)	26 (13.2)	0.371
Education							
High school or above (%)	260 (57.8)	165 (87.3)	24 (12.7)	0.427	155 (79.1)	18 (20.90)	0.095
Employment status (%)				0.407			0.658
Employed	36 (8.0)	26 (10.4)	2 (6.1)		7 (5.4)	1 (3.2)	
Not employed	74 (16.4)	47 (18.9)	4 (12.1)		19 (14.6)	3 (9.7)	
Retirement	340 (75.6)	176 (70.7)	27 (81.8)		104 (80.0)	27 (87.1)	
Monthly income (%)				0.591			0.121
<1,000 RMB	4 (0.9)	3 (1.2)	0 (0.0)		0 (0.0)	1 (3.2)	
1,000–3,000 RMB	34 (7.6)	16 (6.4)	4 (12.1)		9 (6.9)	4 (12.9)	
3,000–5,000 RMB	63 (14.0)	34 (13.7)	5 (15.2)		18 (13.8)	5 (16.1)	
>5,000 RMB	349 (77.6)	196 (78.7)	24 (72.7)		103 (79.2)	21 (67.7)	
Smoking (%)	91 (20.2)	177 (93.9)	2 (6.1)	0.303	172 (87.9)	24 (12.1)	0.226
Drinking (%)	48 (19.7)	175 (92.6)	2 (7.4)	0.497	177 (90.5)	19 (9.5)	0.324
Vintage (months)	46.91 (24.92,88.17)	56.2 (29.8,110.4)	73.3 (38.0,143.7)	0.246	39.5 (22.2,71.9)	33.3 (12.9,58.0)	0.099
Cause of dialysis (%)				0.591			0.711
Diabetic nephropathy (%)	102 (22.7)	4 (1.6)	1 (3.0)		76 (58.5)	20 (64.5)	
Hypertensive nephropathy (%)	69 (15.3)	49 (19.7)	4 (12.1)		13 (10.0)	3 (9.7)	
Glomerulonephritis (%)	141 (31.3)	97 (39.0)	14 (42.4)		25 (19.2)	3 (9.7)	
Polycystic kidney (%)	26 (5.8)	23 (9.2)	1 (3.0)		1 (0.8)	0 (0.0)	
Other	112 (24.9)	76 (30.5)	13 (39.4)		15 (11.5)	5 (16.1)	
Malnutrition (%)	140 (31.1)	151 (80.0)	38 (20.0)	0.026	150 (76.7)	47 (23.3)	0.055
CCI	4.02 ± 1.70	3.22 ± 1.31	4.13 ± 1.87	0.030	4.49 ± 1.64^a^	5.37 ± 1.81^b^	0.003
Number of medications (n)	4.65 ± 2.5	4.32 ± 2.4	4.24 ± 2.3	0.867	5.27 ± 2.5	5.33 ± 3.0	0.921
Antihypertensive (%)	357 (79.3)	191 (76.7)	23 (69.7)	0.376	112 (86.2)	26 (83.9)	0.540
Antidiabetic Agents (%)	102 (22.7)	3 (1.2)	2 (6.1)	0.135	80 (61.5)	16 (51.6)	0.370
Lipid-lowering agents (%)	171 (38.0)	83 (33.3)	14 (42.4)	0.012	57 (43.8)	14 (45.2)	0.883
Anticoagulant (%)	421 (93.6)	237 (95.2)	31 (93.9)	0.021	120 (92.3)	27 (87.1)	0.491
Cardiac drugs (%)	109 (24.2)	47 (18.9)	13 (39.4)	0.005	34 (26.2)	14 (45.2)	0.099
Physical activity (min/day)							
LPA (min/day)	58.35 ± 49.60	495.56 ± 117	459.43 ± 114.30	0.155	458.86 ± 130.83	453.17 ± 121.83	0.817
MVPA (min/day)	58.35 ± 49.60	72.82 ± 53.9	52.3 ± 56.31	0.820	51.01 ± 43.74^a^	30.21 ± 26.92	<0.001
Laboratory parameters							
Hemoglobin (g/dL)	110.6 ± 16.74	114.34 ± 15	113.32 ± 17.66	0.757	107.41 ± 17.79	106.93 ± 15.28	0.872
Albumin (g/L)	39.67 ± 3.54	40.31 ± 3.11	39.78 ± 3.70	0.442	39.01 ± 3.81	39.40 ± 3.62	0.635
PTH (pg/dL)	368.95 ± 305.94	291.67 ± 348.64	564.48 ± 410.49	0.026	334.54 ± 243.49^a^	312.35 ± 240.68	0.6
Uric acid (mmol/L)	19.62 ± 8.51	17.92 ± 6.89	18.25 ± 12.48	0.846	21.28 ± 9.26	20.29 ± 7.39	0.521
Calcium (mg/dL)	2.20 ± 0.25	2.22 ± 0.23	2.20 ± 0.25	0.714	2.18 ± 0.277	2.23 ± 0.28 ^b^	0.253
Phosphorus (mg/dL)	1.93 ± 0.63	2.0 ± 0.65	2.09 ± 0.53	0.535	1.82 ± 0.59	1.99 ± 0.69	0.098
Kt/v	1.34 ± 0.32	1.37 ± 0.31	1.32 ± 0.42	0.437	1.32 ± 0.319	1.28 ± 0.29	0.461
URR	60.48 ± 22.12	58.52 ± 24.45	57.05 ± 23.39	0.785	62.66 ± 19.30	61.07 ± 22.52	0.646

BMI, body mass index; CCI, Charlson Comorbidity Index; LPA, light physical activity; MVPA, moderate-to-vigorous physical activity; PTH, parathyroid hormone; Kt/V, fractional clearance index for urea; URR, urea reduction ratio.

Data are presented as mean ± SD or *n* (%).

^aIn non-depression patients, the Diabetes group vs. the Non-Diabetes group, p < 0.05.^

^bIn depression patients, the Diabetes group vs. the Non-Diabetes group, p < 0.05.^

We then performed trend test analyses of the association between quartiles of moderate-to-vigorous physical activity and depressive symptoms in non-diabetic and diabetic hemodialysis patients (Table 2). In the fully adjusted model, quartiles of moderate-to-vigorous physical activity were negatively associated with depressive symptoms in the diabetic group (OR = 0.076, 95%CI = 0.006–0.95, *p* < 0.05), whereas quartiles of moderate-to-vigorous physical activity were positively associated with depressive symptoms in the non-diabetic group (OR = 1.335, 95%CI = 0.24–7.43, *p* = 0.742). In addition, we analyzed the correlation between light physical activity depressive symptoms, and there was no correlation between light physical activity in both non-diabetes (OR = 1.057, 95%CI = 0.19–5.37, *p* = 0.948) and diabetes (OR = 0.753, 95%CI = 0.13–4.14, *p* = 0.745) in the full adjusted model.

**Table 2:** Regression analysis of moderate-to-vigorous physical activity and depressive symptoms in the non-diabetic and diabetic hemodialysis.

	Crude		Adjusted		Crude		Adjusted	
	OR (95%CI)	*p*-value	OR (95%CI)	*P*-value	OR (95%CI)	*P*-value	OR (95%CI)	*P*-value
MVPA								
Non-diabetes (*n* = 213)					Diabetes (*n* = 237)			
Q1	ref		ref		ref		ref	
Q2	1.495 (0.577,3.874)	0.407	3.677 (0.943,14.342)	0.061	0.343 (0.121,0.971)	0.044	0.354 (0.089,1.408)	0.141
Q3	0.474 (0.146,1.542)	0.215	1.670 (0.360,7.736)	0.512	0.379 (0.141,1.024)	0.056	0.313 (0.080,1.232)	0.097
Q4	0.425 (0.139,1.298)	0.133	1.335 (0.240,7.432)	0.742	0.103 (0.013,0.833)	0.033	0.076 (0.006,0.955)	0.046
*P* trend		0.291		0.223		0.012		0.040
LPA								
Non-diabetes (*n* = 213)					Diabetes (*n* = 237)			
Q1	ref		ref		ref		ref	
Q2	1.613 (0.565,4.602)	0.371	5.488 (1.181,25.501)	0.030	1.075 (0.366,3.155)	0.895	0.925 (0.212,4.043)	0.918
Q3	1.042 (0.339,3.179)	0.943	1.871 (0.354,9.895)	0.461	1.334 (0.483,3.687)	0.578	1.335 (0.336,5.298)	0.682
Q4	0.822 (0.260,2.592)	0.737	1.057 (0.198,5.637)	0.948	0.741 (0.230,2.394)	0.617	0.753 (0.137,4.145)	0.745
*P* trend		0.849		0.857		0.435		0.472

Data are presented as odds ratios, 95% confidence intervals, and *P*-value.

Adjust I model adjust for: Age; Sex; BMI; education level; living alone; widowed; employment status; monthly income; number of drugs; type of drugs; smoking; drinking; cause of dialysis; Kt/v; CCI. BMI, body mass index; CCI, Charlson Comorbidity Index.

## 4 Discussion

The present results showed that the accelerometer-measured physical activity (MVPA) and T2DM were significantly associated with depressive symptoms. In addition, we also found that more MVPA but not LPA is significantly associated with lower depressive symptoms in hemodialysis patients with comorbid T2DM. Further analysis revealed that MVPAQ4 were negatively associated with depression in hemodialysis patients with comorbid T2DM. This finding has important implications for interventionalists and public health practitioners in designing effective depression prevention programs for hemodialysis patients with comorbid T2DM.

In our study, the prevalence of depressive symptoms using the PHQ-9 was 17.2%, which is consistent with other studies. Pretto et al. (2020) and Wang et al. (2017) reported that the prevalence of depressive symptoms in patients undergoing hemodialysis was 14.4 and 21.4%, respectively. However, some studies have shown high prevalence of depression among hemodialysis patients. There are significant disparities in the prevalence of depression among patients receiving hemodialysis in previous literature. For example, a recent cohort study that follow-up time of 4 years did not observe an association between LPA or MVPA and incident depressive symptoms (Konopka et al., 2022; Schuch et al., 2019). There are significant disparities in the prevalence of depression among patients receiving hemodialysis in previous literature, mainly due to differences in the characteristics of the study population, the timing of initiation of hemodialysis treatment and the tools used to screen for depression.

Our study revealed that depressive symptoms were associated with physical activity, including MVPA. The discrepant results between our study and others might be explained by differences in the collection of physical activity data, as well as in the assessment of depressive symptoms. Studies using self-reported physical activity data may be subject to recall bias, which would most likely result in differential misclassification of physical activity data (Althubaiti, 2016). Hemodialysis patients had poor physical performance which may decrease physical activity and also cause depressive symptoms. Cavallo et al. (2022) failed to find the relationship among physical activity measured by accelerometer with mental health in the dialysis. This may be due to different assessment criteria for mental health and different dialysis treatment or populations. To date, rare study focus on the relationship between MVPA and depression in the hemodialysis population with T2DM, which encourages further research to identify possible common underlying determinants of these two conditions.

Moreover, the study suggested that T2DM in hemodialysis patients was nearly 2-3fold risk associated with depressive symptoms, which are in line with some previous studies (Bianchi and Volpato, 2016). Several recent studies have found that physical activity capacity in patients with comorbid T2DM is strongly associated with poor lifestyle and poor health outcomes in hemodialysis patients, such as increasing their risk of developing depressive symptoms (Kim et al., 2022; Dunkler et al., 2015; Tao et al., 2024). Furthermore, we found that hemodialysis patients in the T2DM group had poorer physical activity as measured by accelerometer compared to the non-T2DM group (*p* < 0.05). However, conflicting results have also been reported (Alshelleh et al., 2022; Wang et al., 2017). The inconsistent results are likely due to the small sample size in a single dialysis center and incomplete assessment measures of diabetes status in previous studies. Moreover, the etiology of dialysis-related that reduced physical activity is multifactorial and is associated with biological mechanism. Lewis et al. (2015) demonstrated that skeletal muscle mitochondria are important for physical function. The reduction of leg muscle capillaries and a significant decrease in mitochondrial oxidases impair physical activity in hemodialysis patients (Ebert et al., 2021).

To our knowledge, this is the first study that accelerometer-measured the relationship between physical activity, T2DM and depressive symptoms in patients on hemodialysis. Therefore, in the present study, we found a significant association between MVPA and depressive symptoms in hemodialysis patients with comorbid T2DM. First, the significant association with low physical activity in patients with T2DM. Secondly, the mean MVPA was longer in the T2DM group than in the non-T2DM group (51.0 vs. 72.8, *p* < 0.05) and was less in the T2DM group than in the non-T2DM group (458.8 vs. 495.5). The current evidence for an association between T2DM and physical activity is controversial (Yu et al., 2024). However, the mechanism behind this correlation may be multifactorial. The major risk factors for T2DM (hypertension, heart disease, and obesity) are all strongly associated with physical mobility. Meanwhile, physical activity is associated with symptoms of inflammation, oxidative stress and HPA axis function produced by T2DM. In addition, hyperglycemic state was associated with impaired activity function and reduced physical activity, possibly caused by reduced muscle protein synthesis in insulin-deficient muscles (Bianchi and Volpato, 2016), which is also consistent with the results of a previous study (Su et al., 2023). Therefore, it is reasonable to believe that reduced physical activity due to T2DM may be an important risk factor for predisposing to depression.

There is a bidirectional association between T2DM and depression. The somatic cluster may have stronger links with the putative mechanisms underlying the depression inflammation relationship, such as oxidative stress, hypothalamic–pituitary–adrenal axis (HPA) hyperactivity (Fries et al., 2023; Kelly and Ismail, 2015). First, mitochondrial dysfunction in hemodialysis patients produces oxidative stress leading to a prolonged inflammatory state in the body (Cavallo et al., 2022). Moreover, T2DM leads to dysfunction of the HPA axis, which increases cortisol levels (Alshelleh et al., 2022). And it activates NLRP 3 inflammasome, accelerates interleukin-1β (IL-1β) maturation and secretion, and activates depressed proinflammatory cytokines and their receptors (Ebert et al., 2021). Therefore, in our study, Abnormalities in depressive states in diabetic dialysis patients may precede and play a greater role in the increase in somatic symptoms.

In addition, we found that depression with a more significant correlation with the Q4 of MVPA quartiles. Even after adjusting for potential confounders, MVPAQ4 remained negatively associated with depressive symptoms. Therefore, the inclusion of physical activity in hemodialysis treatment for comorbid T2DM, reducing the effects of hyperglycemia, and having a positive effect on their disease management (Fedewa et al., 2017). In addition, exercise can stimulate the reconstruction of neural structures related to depression and reduce chronic inflammation (Arida et al., 2009; Felez-Nobrega et al., 2021). Meanwhile, physical exercise improves the function of the neuroendocrine system, increases the breakdown of anabolic muscle components and improves body functions (Kim et al., 2022; Wiles et al., 2012). In addition, habitual moderate-intensity physical activity in hemodialysis patients can also reduce the side effects of medications taken (Manea et al., 2012). In the future, much greater attention must be paid to mental health during the renal rehabilitation, especially in depressed patients with comorbid T2DM, with early intervention for depressive symptoms.

### 4.1 Strengths and limitations

We would like to emphasize several strengths of our study. First, it is the first multicenter study to examine the relationship between physical activity and depressive symptoms among hemodialysis patients across different diabetic states. Second, while most previous studies used self-reported physical activity measures, which are prone to recall and social desirability biases, we used accelerometers that monitor physical activity of different intensities continuously through 24-h days. Furthermore, the major advantage of the ActiGraph GT3X+ is that it distinguishes changes in posture based on precise acceleration information so that MVPA and physical activity can be measured more accurately than other monitors. Moreover, it is more comfortable and has no foreign body feeling compared to the belt. And it can more accurately measure the daily physical activity modifications of hemodialysis patients. Third, our cutoffs for activity categories and round definitions are based on widely used protocols, which enhances comparability with previous studies.

Nonetheless, this study is not without limitations. First, the wrist-worn accelerometers could overestimate PA levels, so their physical activity patterns may be higher than those of normal exercise habits. Second, external factors, such as family problems, dietary habits, and environmental changes, may influence physical activity. Finally, the cross-sectional nature of the data in this study prevents us from drawing conclusions about causality, and all of the samples in this study were from one city, which resulted in a limited sample size and some regional limitations. In the future, we will continue to expand the geographical scope and increase the time span.

## 5 Conclusion

In this study, we found that accelerometer-measured MVPA in patients with T2DM combined with hemodialysis was associated with depression. In addition, further analyses demonstrated the relationship between Q4 in MVPA quartiles and depression in the T2DM group. The causal relationship between increased moderate-to-vigorous physical activity and depressive symptoms in patients with type 2 diabetes requires further study.

## Data Availability

The original contributions presented in the study are included in the article/supplementary material, further inquiries can be directed to the corresponding author.
